# Synthesis, Docking, Computational Studies, and Antimicrobial Evaluations of New Dipeptide Derivatives Based on Nicotinoylglycylglycine Hydrazide

**DOI:** 10.3390/molecules25163589

**Published:** 2020-08-07

**Authors:** Hemat S. Khalaf, Ahmed M. Naglah, Mohamed A. Al-Omar, Gaber O. Moustafa, Hassan M. Awad, Ahmed H. Bakheit

**Affiliations:** 1 Chemistry Department, College of Science and Arts, Jouf University, Al Qurayyat 77425, Saudi Arabia; hematsalama2016@gmail.com; 2 Photochemistry Department, Chemical Industries Research Division, National Research Centre, Dokki, Cairo 12622, Egypt; 3 Drug Exploration and Development Chair (DEDC), Department of Pharmaceutical Chemistry, College of Pharmacy, King Saud University, Riyadh 11451, Saudi Arabia; malomar1@ksu.edu.sa; 4 Peptide Chemistry Department, Chemical Industries Research Division, National Research Centre, Dokki, Cairo 12622, Egypt; gosman79@gmail.com; 5 Nahda University, New Beni-Suef City, Beni-Suef 62521, Egypt; 6 Chemistry of Natural and Microbial Products Department, Pharmaceutical Industries Division, National Research Centre, Dokki, Cairo 12622, Egypt; awadmmhassan@gmail.com; 7 Department of Pharmaceutical Chemistry, College of Pharmacy, King Saud University, Riyadh 11451, Saudi Arabia; abujazz76@gmail.com; 8 Department of Chemistry, Faculty of Science and Technology, Al-Neelain University, Khartoum 12702, Sudan

**Keywords:** docking, covalent docking, computational studies, anti-microbial peptides, biological evaluations, nicotinoyl-glycyl-glycine-hydrazide

## Abstract

Within a series of dipeptide derivatives (**5**–**11**), compound **4** was refluxed with d-glucose, d-xylose, acetylacetone, diethylmalonate, carbon disulfide, ethyl cyanoacetate, and ethyl acetoacetate which yielded **5**–**11**, respectively. The candidates **5**–**11** were characterized and their biological activities were evaluated where they showed different anti-microbial inhibitory activities based on the type of pathogenic microorganisms. Moreover, to understand modes of binding, molecular docking was used of Nicotinoylglycine derivatives with the active site of the penicillin-binding protein 3 (PBP3) and sterol 14-alpha demethylase’s (CYP51), and the results, which were achieved via covalent and non-covalent docking, were harmonized with the biological activity results. Therefore, it was extrapolated that compounds **4**, **7**, **8**, **9**, and **10** had good potential to inhibit sterol 14-alpha demethylase and penicillin-binding protein 3; consequently, these compounds are possibly suitable for the development of a novel antibacterial and antifungal therapeutic drug. In addition, in silico properties of absorption, distribution, metabolism, and excretion (ADME) indicated drug likeness with low to very low oral absorption in most compounds, and undefined blood–brain barrier permeability in all compounds. Furthermore, toxicity (TOPKAT) prediction showed probability values for all carcinogenicity models were medium to pretty low for all compounds.

## 1. Introduction

Nicotinic acid, known as niacin, has been used since 1950 as a lipid-altering therapy [1]. Nicotinic acid also reduces triglyceride and lipoprotein cholesterol levels [2]. Also, it reduces the risk of atherosclerotic coronary heart disease, atherosclerotic progression, and cardiovascular complication factors [3,4,5]. Nicotinic acid derivatives, as well as their isomers processes such as antibacterial, antioxidant, anti-inflammatory, anticarcinogenic, antitubercular activities, cell signaling, and regulating gene expression, are involved in the synthetic pathway of lipids [6,7,8]. Otherwise, there are several research articles in the field of synthetic peptide chemistry focusing on nicotinic acid’s anti-microbial properties for therapeutic applications. Anti-microbial peptides (AMPs) are a very interesting class of antibiotics distinguished by their unique antibiotic action and their decreasing tendency for improving resistance compared to antibiotic drugs [9]. AMPs can be used as delivery vectors for many bioactive candidates and have been shown to kill Gram-positive and Gram-negative bacteria [10]. Additionally, AMPs can kill enveloped viruses and fungi [11]. Furthermore, they have many modes of action for kill microbes, [12] and the modes of action are likely to vary across several bacterial species [13]. A portion of anti-microbial peptide derivatives eliminates bacteria and fungi, such as cases where Psoriasin kills *E. coli* alongside other filamentous fungi [14].

Covalent inhibitors possess a group of reactive functions that can form a covalent bond with a target, which represents the spearhead in this type of docking. Over the last decade, the design of medicines, which can form a joint covalent bond with drug targets, has become more interesting [15]. Almost 30% of the drugs marketed target enzymes that are reacted via covalent addition, therefore targeted covalent inhibitors (TCI) are becoming increasingly significant [16]. Covalent inhibitors have been extensively studied in terms of pharmacological advantages and covalent inhibitors were shown to achieve longer residence periods than non-covalent inhibitors [17] and to increase target selectivity [18,19].

For modeling studies, PBP isoform 3 and CYP51 were chosen as the target protein; furthermore, Penicillin-binding proteins (PBPs) are minority components that were found in the particular structures of bacteria cell envelopes. These proteins are specific enzymes catalyzing peptidoglycan biosynthesis in the latter phase. The PBP3 is one from all Penicillin-binding proteins (PBPS) of *Escherichia coli*, which has proven participation in cell septation. PBP3 was affected by mutations of the proteins’ selective inhibition through lactams inducing the cells of *E. coli* to form filaments. PBP has received much attention due to the clear involvement of PBP3 in the cell division. PBP3 potentially acts as an oligomeric enzyme being part of a multimeric complex, which may in turn help to modulate this PBP’s activity [20]. More than two of six PBPs, which are PBP1a, PBP1b, PBP2a, PBP2b, PBP2x and PBP3, that are highly preserved are present in any bacterial species. This the major pathogen of humans causing upper respiratory tract (URT) infections that are responsible for deaths of more than 1.6 million yearly [21]. Although in the cytochrome p450 superfamily, CYP51 is used as a major drug target [22]. Sterol 14α-demethylase (CYP51) is the most functionally conserved cytochrome P450 (CYP) monooxygenase. One of the important functions of sterol 14α-demethylase (CYP51) is protected by the cytochrome P450 (CYP) monooxygenase. In all biological kingdoms, CYP51 orthologs are located and united into one (CYP51) family (despite very low identity sequence of amino acid across phylogeny) [23]. Depending on these observations and in continuation of our previous studies for anti-microbial and anticancer agents [24,25,26,27,28,29,30,31,32,33,34,35,36,37,38,39,40,41], this study was aimed at synthesizing a series of novel dipeptide compounds that were hypothesized to affect systemic anti-microbial measurements.

## 2. Results and Discussion

### 2.1. Chemistry

**Scheme 1:**
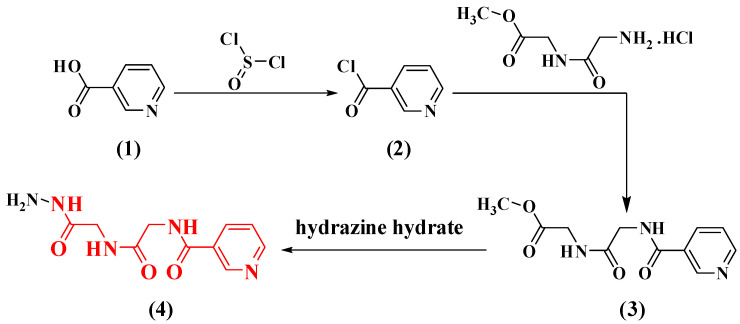
Synthetic routes for nicotinoyl-glycyl-glycine-hydrazide (**4**)**.**

Novel dipeptide derivatives based on nicotinoyl-glycyl-glycine-hydrazide were synthesized in this study; these derivatives are hypothesized to contain different antifungal and antibacterial characteristics. The synthesis of *N*-(2-((2-hydrazinyl-2-oxoethyl)amino)-2-oxoethyl)nicotinamide (**4**) is the result of the transformation of nicotinic acid (**1**) to nicotinoyl chloride (**2**) due to its reaction with thionyl chloride**.** The latter compound was then paired with glycyl-glycine methyl ester to yield nicotinoyl-glycyl-glycine-methyl ester (**3**) and hydrazinolysis of compound (**3**) with alcoholic hydrazine hydrate, which afforded the corresponding dipeptide hydrazides (**4**) (Scheme 1).

**Scheme 2:**
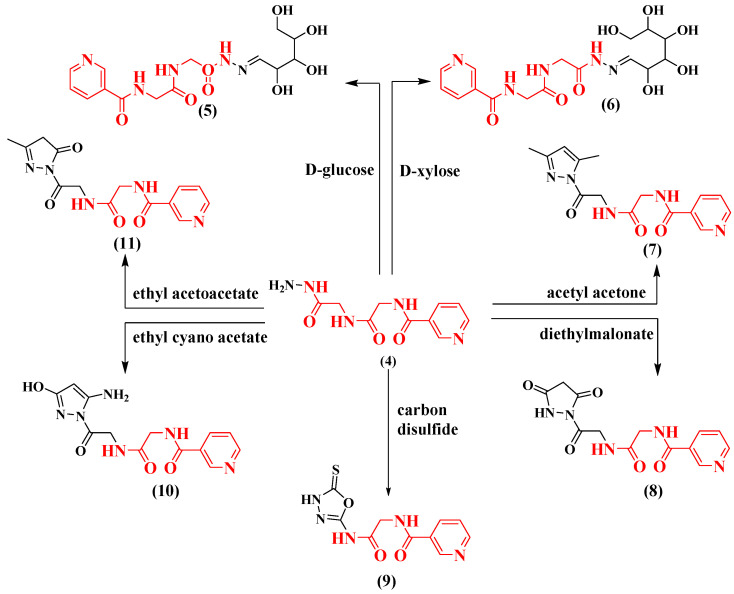
Synthetic routes for nicotinoyl-glycyl-glycine-hydrazide derivatives **5–11.**

Condensation of compound (**4**) with d-glucose or d-xylose took place by refluxing it in absolute ethanol in the proximity of acetic acid, thus producing the paralleling hydrazides **5** and **6**. On the other hand, when nicotinoyl-glycyl-glycine-hydrazide **4** reacted with acetylacetone and diethylmalonate, giving compounds **7** and **8**, the consuming time of refluxing was 12 h and 5 h, respectively. Furthermore, compound **4** reacted with carbon disulfide, giving oxadiazole derivatives **9**. A mixture of compound **4** and ethyl cyanoacetate and ethyl acetoacetate were refluxed in ethanol, giving *N*-(2-(2-(5-amino-3-hydroxy-1*H*-pyrazol-1-yl)-2-oxoethylamino)-2-oxoethyl) nicotinamide a *N*-(2-(2-(3-methyl-5-oxo-4, 5-dihydro-1*H*-pyrazol-1-yl)-2-oxoethylamino)-2-oxoethyl) nicotinamide **10** and **11,** respectively (Scheme 2).

### 2.2. Evaluation of Biological Aspects

The antibacterial and antifungal process of the novel synthesized compounds **5**–**11** and the initial compound, nicotinoyl-glycyl-glycine-hydrazide **4** ware compared with a panel of pathogenically assessed organisms in terms of anti-microbial and antifungal processes, demonstrated in Table 1. The outcomes revealed that a part of these compounds was extremely active in terms of biology in association with different activities on the spectrum.

The information in Table 1 shows various anti-microbial influences against every tested pathogenic microorganism. Concerning *B. subtilits*, nicotinoyl-glycyl-glycine-hydrazide **4** showed a strong inhibitory effect and recorded 29 mm. On the other hand, hydrazinepyrazole and oxadiazole derivatives **7** and **9** showed a moderate inhibition effect of 18 and 19 mm while the other assayed compounds were recorded showing a weak inhibition effect compared with the antibacterial drugs. Similarly, most *E. coli* samples recorded a moderate inhibition effect except sample compound **4** which showed a strong inhibitory effect and recorded 30 mm when cross-referenced with the used standard antibiotic in the current study.

Regarding *C. albicans*, compound **4** showed a strong inhibitory effect and recorded 28 mm, while compounds**10** and **11** recorded a weak inhibition effect. On the other hand, samples **5**–**9** exhibited a moderate inhibitory influence within 16–20 mm with reference to the standard used antifungal in the current study. Regarding *A*. *niger,* a pathogenic fungus, most of the compounds have an adverse influence against this pathogen albeit compounds **4** and **8** maintained a moderate inhibition effect compared with the antifungal drugs.

### 2.3. Computational Studies

#### 2.3.1. Active Site

In the penicillin-binding protein 3, Serine (Ser392) of the active site faces an approximately 20 Å deep and 15 Å large groove running along strand β3, allowing peptidoglycan and/or β-lactams entry. β3 contains the KS/TG motif (residues 618–621). Between two helices and the catalytic serine, the SXN motif, which the active serine (motif SXN) is situated in a loop between helices α4 and α5, (residues 448–450) takes place. The interaction of these three motifs takes place by hydrogen bonds, such as Lys618 hydrogen bonds to the Ser448 side chain and Ser392 main chain oxygen in the apo form. Furthermore, the hydrogen bonds were formed with the Ser392 side chain and with Lys395 at the other motifs SXXK, which is the serine of active is located at the beginning of helix α2 and is followed by a lysine to form a SXXK motif. Although the active site is available, it is occluded more than in (PBP4) *E. coli*, which is easily inhibited by a synthesized compound such as β-lactams drugs.

The cavity of the active site of sterol 14α-demethylase includes a molecule of water, which is coordinated to the iron of a heme group, in the substrate-free state. Evidently, this molecule of water is strongly bound with iron and it is not completely replaced by the substrate residual of this water, which is found close to the iron, partaking in the delivery of catalytic proton. The derivatives of heterocyclic compounds, which consist of a single or multi basic atom, and such as triazole, imidazole, pyridine, or our synthesized compounds, react as a stronger ligand for the heme iron. However, these compounds formed a coordination complex with the heme, which readily replaces the molecule of water and that affect in the binding of substrate and metabolism [42]. Binding an inhibitor does not result in wide-ranging conformational rearrangements, but it induces unexpected local modifications in the active site, such as the formation of hydrogen-bonding, which links two remote functionally important protein segments through the amide group of fragment inhibitor and alters the environment of the heme group.

#### 2.3.2. Docking Study

For better optimization of the compounds in this study, the docking protocol performed in the molecular operating environment (MOE) was used to dock all the hits previously obtained into the binding site of PBPs and 14-alpha sterols. Before applying the initial docking protocol, the ligand was extracted from the complex structure of the protein crystal, and then re-docked into the binding site of the protein, thereby validating the mooring protocol. The root mean square deviation (RMSD) among the conformation of co-crystallized and re-docked was evaluated via using MOE’s SVL script (which is programming language built into MOE.) and shown to be 1.86 and 169 Å, for PBP3 and CYP51, respectively; this docking protocol was observed to be successful in reproducing the experimentally calculated mode of binding for the respective complex of the protein-ligand. Therefore, the binding modes of the other compounds could be studied by using the MOE docking protocol and the parameter set.

**Figure 1:**
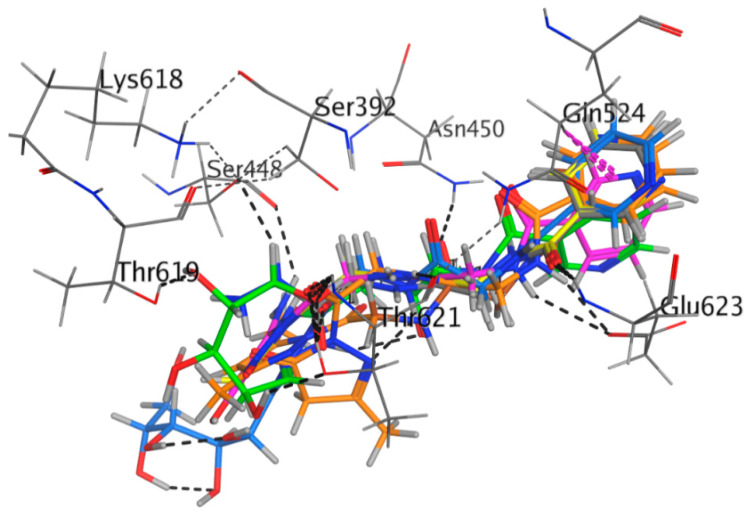
Compounds **4** to **11** superimposed by their non-covalent-docking poses in the active site of PBP3 pocket.

**Figure 2:**
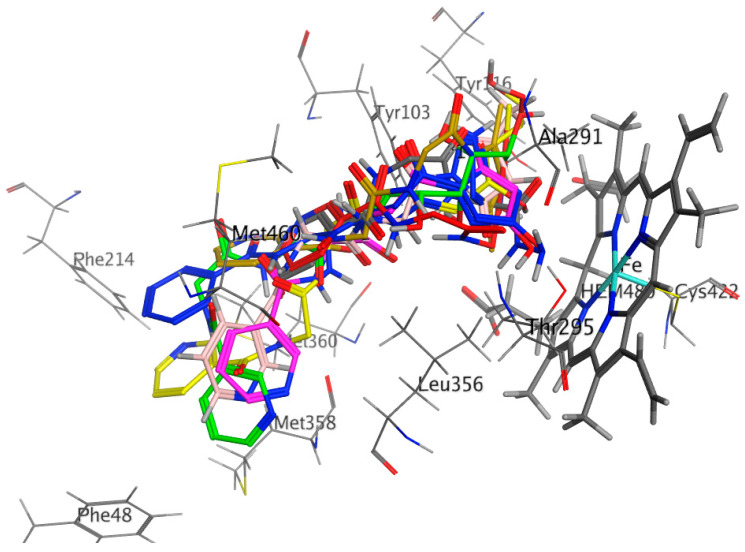
Compounds **4** to **11** superimposed by their poses of non-covalent docking in the active site of CYP51 pocket.

**Figure 3:**
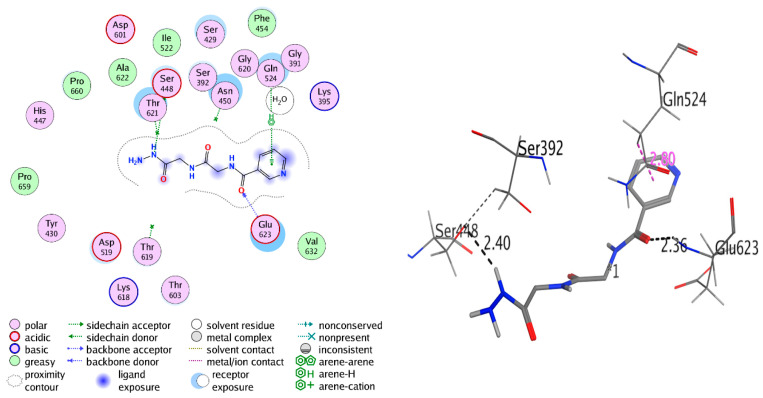
2D and 3D interaction of compound **4** with 3VSL.

**Figure 4:**
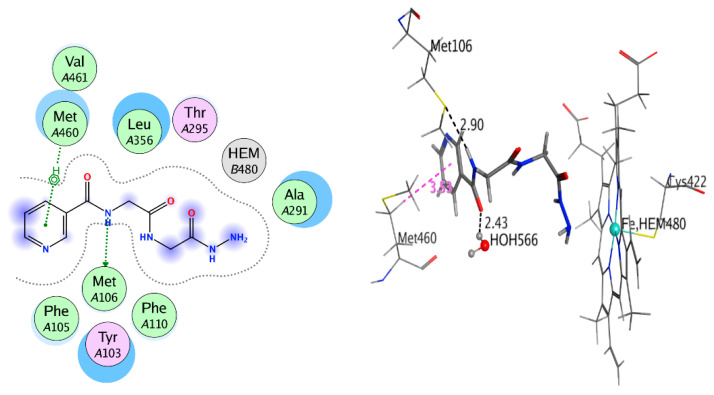
2D and 3D interaction of compound **4** with 3GW9.

For the synthesized compounds **4**–**11**, Negram (NA), Streptomycin (S), and Neomycin (N) as biological test references were docked into the penicillin-binding protein 3 (PBP3) and sterol 14-alpha demethylase’s (CYP51) active sites using a MOE software package to interpret the interactions of binding; see Table 2 and Table 3. In the docking studies, the selected pose for of all synthesized compound references showed significant binding interactions inside the active pocket with amino acid residues Phe48, Tyr103, Tyr116, Phe214, Ala291, Thr295, Leu356, Met358, Met360, Cys422, Met460, and Val461 for the 3GW9 and Ser392, Lys395, Ser429, Ser448, Asn450, Thr619, Thr621, Glu623, Val632, Pro659, and Pro660 for 3VSL (Figure 1 and Figure 2). The hydrogen-bonding interactions with amino acid residues are tabulated below for all compounds. The derivatives of Nicotinoylglycine showed docking values from −9.597 to −17.891, while the binding affinity ranged from −4.781 to −7.152 kcal/mol (Table 2). For more detail, compound **4**, which was a ethane hydrazide substitution and one of the top-ranked docked conformations, showed good binding affinity (−5.315 Kcal/mol), and perfect docking score (−11.868) as well as showed the hydrogen bonds with Ser448, Glu623, and Gln524 residues with acceptable bond length in range (2.3–2.80 Å) (Figure 3 and Figure 4). However, compound **4**, which interacted with a hydrophobic pocket of sterol 14-alpha demethylase, showed strong binding affinity (–5.886 kcal/mol) and good docking score (–9.333) as well as forming a hydrogen bond in the active site with Ser448, Glu623, and Gln524 (with bond length less than 3). Therefore compound **4** is one of the top-ranked biological activities besides the docking results.

**Figure 5:**
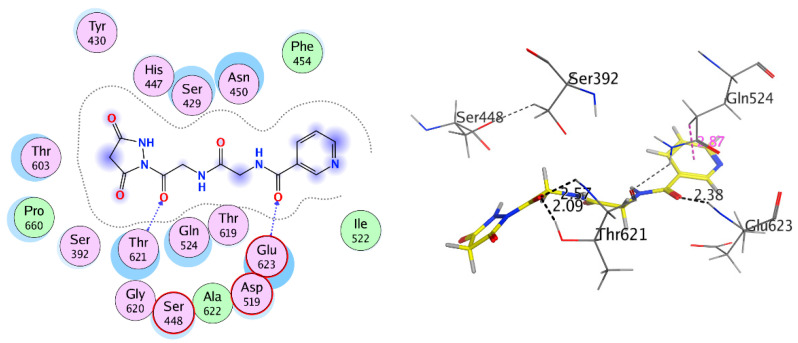
2D and 3D interaction of compound **8** with 3VSL.

**Figure 6:**
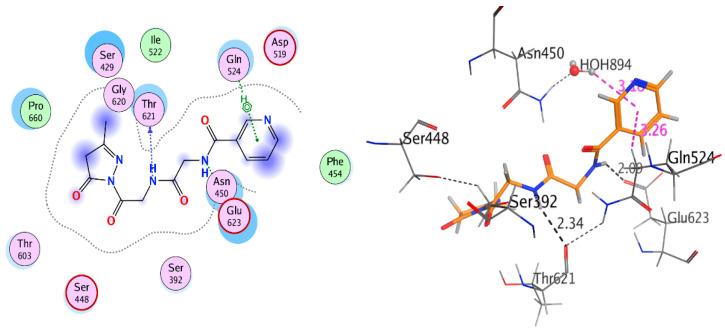
2D and 3D interaction of compound **9** with 3VSL.

On the other hand, the diethylmalonate, acetyl acetone, and carbon disulfide substituted compounds **7**–**9**, which were the top-ranked docked conformations, were integrated into the hydrophobic pocket of the penicillin-binding protein. The binding affinity of the complex of these compounds with the penicillin-binding protein 3 were found to be very good (−4.315 to −5.966 Kcal/mol) and there was a good docking score in the range (−7.344 to −11.84) as well as interactions of the binding of Nicotinoylglycine derivative within the cavity of active site (Figure 5 and Figure 6). Furthermore, the Glycine and substitution parts of these ligands moved to hydrophilic part of the cavity of active site such as Serine (Ser), Threonine (Thr), Tyrosine (Tyr), Glutamine (Gln) Asparagine (Asn), and Histidine (His), therefore being bonded via hydrogen bond with one or more of these residues. For example, compounds **7, 8,** and **9** interacted with the oxygen of carbonyl or nitrogen of amide group from the Glycine part via hydrogen bond in active site with Glu623 Asn450, Gln524, Thr621, and Ser448 with bond length less than 3 Å (Figure 5 and Figure 6). All these results illustrated the good result of biological activities tests.

**Figure 7:**
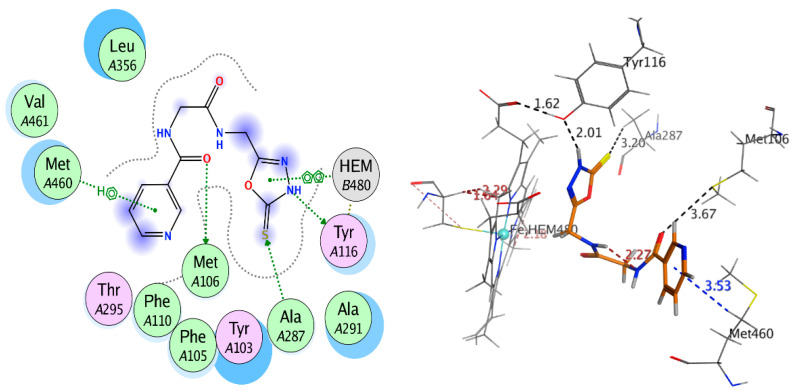
2D and 3D interaction of compound **8** with 3GW9.

**Figure 8:**
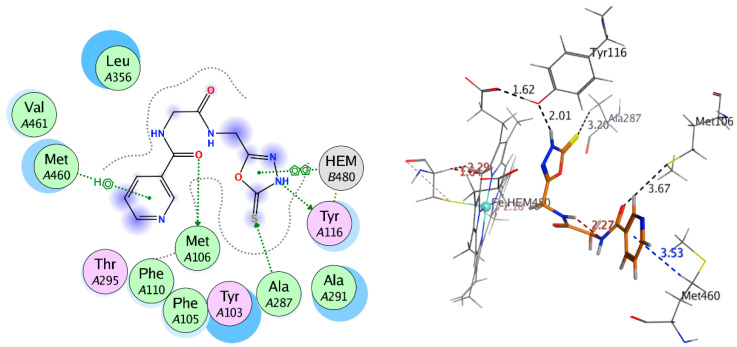
2D and 3D interaction of compound **9** with 3gw9.

The synthesized compounds bonded with CYP51 via hydrophobic interactions and H-bonding with the residues of active site that are including the amino acid residues Phe48, Tyr103, Tyr116, Phe214, Ala291, Thr295, Leu356, Met358, Met360, Cys422, Met460, and Val461, within 6 Å from that ligands (Figure 2). The most distinct feature of the bound Nicotinoylglycine derivative is that pyridine rings for this derivative located away from heme group (Figure 2). In addition, the other terminal of molecule is showing more polar and which is located close by the heme group and thereby play a role of a substrate recognition. The polar terminal of compounds **8**, **9**, and **10** are pyrazolidine-3, 5-dione, oxadiazole-thione and 3-amino-pyrazol-5-ol, which are bonded π-bond with one ring in the heme group, this association reduced the distance between the ligands and the heme group, and thus showed a good explanation for the ability of these compounds to inhibit the CYP51 enzyme (Table 3, Figure 7 and Figure 8). Based on data of biological activity and docking results, it was deduced that compounds **4**, **7**, **8**, **9**, and **10** had the good potential to inhibit sterol 14-alpha demethylase and penicillin-binding protein 3.

#### 2.3.3. Covalent Docking

The derivatives of Nicotinoylglycine achieved good docking scores for most synthesized compounds, supported by the results of the Inhibition Zone testing (Table 2). The PBP3 is one of the key targets of bacteria for irreversible inhibitors. Here the covalent docking studies for these Nicotinoylglycine derivatives observed that newly compounds could have been a potential target of PBP3. These compounds have an active double bond, which is interacted as an acetalization reaction of aldehyde or ketone, could be attacked by nucleophiles. Therefore, based on electrophilic existence in these Nicotinoylglycine derivatives, we suggested using an irreversible mechanism similar to the penicillin inhibitors to inhibit PBP3. Furthermore, all synthesized ligands were simulated covalently into the PBPs catalytic pocket. Table 3 lists the predicted docking scores, binding affinity, bond type, bond length, and residues, which interacts with ligands. All the Nicotinoylglycine derivatives covalently interacting with PBPs active site showed favorable docking scores ranging from –9.043 to 012.996 and binding affinity is about –5.407 to –6.792 kcal/mol Table 4.

**Figure 9:**
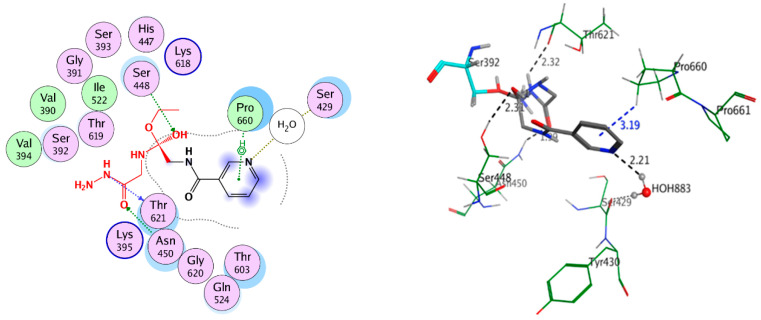
2D and 3D covalent interaction of compound **4** with 3VSL.

**Figure 10:**
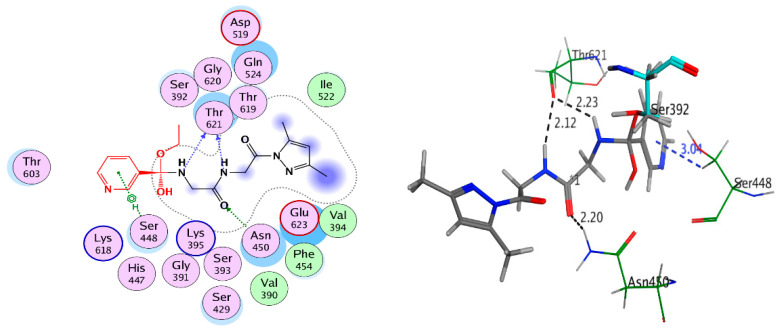
2D and 3D covalent interaction of compound **8** with 3VSL.

The docked poses of compounds **4**–**11** showed that interacted covalently with Ser392 residue in active site of the PBP3; moreover, it was interacted by hydrophobic interaction and hydrogen bond with the side chain residues in the cavity of active site. In the active site, compound **4** formed five hydrogen bonds with amino acid residues Asn450, Ser448, Thr621, and Pro660 besides HOH883 with bond length not more than 3.12 Å, Figure 9. However, compounds **8**, **9**, **10**, and **11** formed three hydrogen bonds or more with Asn450, Thr621, and Ser448 with bond length less than 3.00Å; see Figure 10. The covalently docking results could explain the good biological activity results for compounds **4**, **7**, **8**, **9**, **10**, and **11**.

##### Properties of Absorption, Distribution, Metabolism, Excretion, and Toxicity (ADMET)

The ADMET was predicted by the analysis of various descriptors and pharmaceutical properties via studio discovery biovia, and then the resulting data is summarized in Table 5. Furthermore, different parameters and drug-like characteristics, which are based on Lipinski’s rule of five for all compounds, were analyzed and achieved good results in most of them. The unacceptable results that were found for the specific range were achieved in compounds **5** and **6**.

The drug is described to be ideal when its absorption is rapid and complete from the gastrointestinal tract, is especially distributed in the body to the site of action, is metabolized without immediately removing its activity, and is suitably eliminated from body without any damage. Therefore, the quality of the compounds for human therapeutic use, and the properties of pharmacokinetic for example ADMET is a significant determinant [43,44,45,46]. The computation studies of pharmacokinetic properties are performed based on chemical structures of potential drugs, and several descriptor types have been proposed. Parameters of adsorption comprise Lipinski’s rule of 5 that require that logarithms of partition coefficients for octanol-water (logP) must be equal or less than 5; molecular weight (MWt) for all compounds are found to be less than 450 g/mol; and the number of hydrogen bond acceptors (HBA) are fewer than 8 in all compounds except compounds **5** and **6**. The number of hydrogen bond donors (HBD) were less than 5 in all compounds except compounds **5** and **6**, also the number of rotatable bonds (Nrot) were less than 10 in all compounds except compounds **5** and **6**. For ionic substances especially, the coefficient of distribution (logD) were less than 3.5 except compounds **5** and **6**. The topological polar surface area (TPSA) were found more than 140 Å^2^ in compounds **5**, **6**, **9** and **10**, while compounds **4** and **11** were shown to be f good value of Polar Surface Area (PSA) thereby indicating good oral bioavailability. In addition, the aqueous solubility (Sw) was found to be greater than 0.010 mg/mL in all compounds. The level of absorption of compounds **7** and **9** is 0 which indicates very good human intestinal absorption, and for compound **11** it is 1 (level 1; moderate). For compounds **4** and **8** it is 2 which is outside of the 99% ellipse (level 2; low). For compounds **5**, **6**, and **10** it is 3 (Level 3; very low). All compounds have very high aqueous solubility levels, such as levels 4 and 5. All compounds have very low or undefined values for BBB penetration levels (level 4). Furthermore, all compounds are predicted to have hepatotoxicity values. Our results indicate that all compounds are toxic to the liver. Similarly, all ligands are satisfactory with respect to the CYP2D6 liver, suggesting that compounds are inhibitors of CYP2D6. This demonstrates that all studied compounds are not well metabolized in Phase-I metabolism. Finally, the ADMET plasma protein binding property prediction remarks that all compounds lay outside of the 95% ellipse, clearly suggesting that all poor compounds have good bioavailability and are poorly bound to carrier proteins in the blood.

##### In Silico Toxicity

To predict the drug potential toxicity, however TOPKAT (Discovery Studio 3.5, Accelrys, Inc., San Diego, CA, USA), which is established in the silico toxicity prediction software tool, was used [47]. The toxicity prediction was performed in terms of probability values, TOPKAT (Toxicity Prediction by Komputer Assisted Technology), which was used accurately, developed and validated Quantitative Structure Toxicity Relationship (QSTR) models. The QSTR models in TOPKAT are progressed for continuous endpoints via analysis of regression and for categorical endpoints via analysis of the discriminant. To improve the models of prediction, TOPKAT uses numerous two-dimensional molecular, electronic, and spatial descriptors. Furthermore, the Optimal Predictive Space (OPS) that was patented during validation of the method was applied via TOPKAT in respect of the assurance estimation of the prediction. Probability values lower than 0.30 are considered to be low probabilities for any toxicological end point, while probability values bigger than 0.70 are known as high probabilities [48]. The probability of the molecule’s toxicity was determined using the reasoning engine [49].

The results of the toxicity profile predicted for drug using TOPKAT are summarized in Table 6. By measuring the values of probabilities, it can be observed that with a few exceptions that the toxicity profile of the drug is fairly similar. All compounds showed high probability values for Skin Irritancy None vs. Irritant, Ocular Irritancy None vs. Irritant and Ocular Irritancy Mild vs. Moderate Severe, while compound **9** showed high probability values for Skin Sensitization None vs. Sensitizer. However, probability values for all carcinogenicity models were medium to pretty low for all compounds.

## 3. Materials and Methods

### 3.1. Chemistry

#### 3.1.1. Materials

The chemicals and organic solvents were procured from Sigma (St. Louis, MO, USA) and Fluka (Buchs, Switzerland). Melting points were deduced via digital electrothermal melting point apparatus in opened glass capillary tubes and without further adjustment. (R_f_) values were determined by using Thin-Layer Chromatography (TLC) and were carried out on silica gel aluminum sheets, 60 F_254_ (E. Merck, Burlington, MA, USA). Elemental microanalyses for carbon, hydrogen, and nitrogen (Micro-Analytical Unit, Cairo University, Egypt) were found to fall within good limits of theoretical values (±0.4%). Infrared (IR) spectra were accounted for by KBr disks using the Fourier transform infrared spectrophotometer, Shimadzu; Model: IR Affinity-1S, at the Micro-Analytical Unit, Cairo University, Egypt. Mass spectra’s dimensions were assessed on gas chromatograph-mass spectrometer, Shimadzu (Shimadzu, Kyoto, Japan); Model: QP2010 ultra, (Micro-Analytical Unit, Cairo University, Egypt). (^1^H NMR and ^13^C NMR) spectra were operated on JEOL, JöEL 500 MHz instruments in (DMSO-*d_6_*).

#### 3.1.2. Synthesis of Nicotinoyl Chloride (2), Nicotinoyl Glycyl-glycine-methyl Ester Hydrochloride (3), and N-(2-((2-hydrazinyl-2-oxoethyl) amino)-2-oxoethyl) nicotinamide (4)

These compounds were composed in accordance with the methods reported beforehand [30,50,51].

#### 3.1.3. The General Procedure for the Synthesis of Compounds **5** and **6**

A blend of (0.01 mol) of compound **4** and d-glucose and/or d-xylose (0.01 mol) in ethanol (50 mL) and acetic acid as a catalytic agent was heated at 80 °C for one hour. The resulting precipitate was filtered in hot temperature conditions and rinsed several times with ethanol to give compounds **5** and **6,** respectively.

***(E)-N-(2-oxo-2-(2-oxo-2-(2-(2, 3, 4, 5-tetrahydroxypentylidene) hydrazinyl) ethylamino) ethyl) nicotinamide* (5).** Yield: 70%; m.p. 206–208 °C, IR (cm^−1^): (KBr): ν = 3423 (NH stretching), 3004 (CH, aromatic), 2917 (CH, aliphatic), 1651, 1431, and 1414 (C=O amide I, II and III, respectively). ^1^H-NMR (500 MHz, δ, ppm, DMSO-*d*_6_*): δ* = 9.6, 8.95 (s, 3H, 3NH, D_2_O exchangeable, amide, hydrazide), 8.80–7.60 (s, 4H, aromatic H, Pyr.), 7.4 (d, 1H, NHCH), 4.8–4.6 (s, 4H, 4OH, D_2_O exchangeable), 4.17, 4.00 (t, 4H, 2CH_2_, α-gly), 3.3–3.1 (d, 3H, 3CH, aliphatic H). MS (EI, 70 eV): *m/z* (%) = 383 (M^+^, 0.59), 345 (0.44), 201 (1.01), 153 (1.04), 129 (4.19), 111 (19.59), 84 (40.17), 59 (100), 55 (81.96), 50 (3.83). Molecular formula (M.wt.): C_15_H_21_N_5_O_7_ (383.4). Calculated analysis: C, 47.00; H, 5.52; N, 18.27; found: C, 47.05; H, 5.55; N, 18.33.

***(E)-N-(2-oxo-2-(2-oxo-2-(2-(2, 3, 4, 5, 6-pentahydroxyhexylidene) hydrazinyl) ethylamino) ethyl) nicotinamide* (6).** Yield: 65%; m.p. 210–211 °C, IR (cm^−1^): (KBr): ν = 3423 (NH stretching), 3002 (CH, aromatic), 2916 (CH, aliphatic), 1651, 1424, and 1315 (C=O amide I, II and III, respectively). ^1^H-NMR (500 MHz, δ, ppm, DMSO-*d*_6_): *δ* = 9.2–9.1 (s, 3H, 3NH, D_2_O exchangeable, amide, hydrazide), 8.82–7.20 (s, 4H, aromatic H, Pyr.), 7.4 (d, 1H, NHCH), 4.7–4.7 (s, 5H, 5OH, D_2_O exchangeable), 3.53 (t, 4H, 2CH_2_, α-gly), 2.95 (d, 4H, 4CH, aliphatic H). MS (EI, 70 eV): *m/z* (%) = 413 (M^+^, 0.23), 378 (0.47), 270 (1.08), 235 (1.10), 149 (4.15), 105 (10.86), 63 (100), 57 (44.21), 55 (36.80), 50 (6.51). Molecular formula (M.wt.): C_16_H_23_N_5_O_8_ (413.4). Calculated analysis: C, 46.49; H, 5.61; N, 16.94; found: C, 46.54; H, 5.65; N, 16.99.

#### 3.1.4. The General Procedure for the Synthesis of Compound **7**

A mixture of nicotinoyl-glycyl-glycine-hydrazide (**4**) (0.01 mol) and acetyl acetone (0.03 mol) in ethanol (20 mL) was refluxed for 12 h. The mixture was concentrated, and the precipitate recrystallized from the dimethylformamide (DMF) to create compound **7.**

***N-(2-(2-(3, 5-dimethyl-1H-pyrazol-1-yl)-2-oxoethylamino)-2-oxoethyl) nicotinamide* (7).** Yield: 82%; m.p. 180–182 °C, IR (cm^−1^): (KBr): ν = 3423 (NH stretching), 3119 (CH, aromatic), 3050 (CH, aliphatic), 1600, 1521, and 1446 (C=O amide I, II and III, respectively). ^1^H-NMR (500 MHz, δ, ppm, DMSO-*d_6_): δ* = 9.18, 9.09 (s, 2H, 2NH, D_2_O exchangeable, amide), 8.86–7.69 (s, 4H, aromatic H, Pyr.), 6.28 (d, 1H, CH, Pyrazole ring), 4.17, 3.85 (t, 4H, 2CH_2_, α-gly), 1.4–1.2 (s, 6H, 2CH_3_). MS (EI, 70 eV): *m/z* (%) = 315 (M^+^, 0.58), 241 (1.00), 176 (5.74), 123 (100), 105 (80.77), 78 (69.77), 50 (24.93). Molecular formula (M.wt.): C_15_H_17_N_5_O_3_ (315.3). Calculated analysis: C, 57.13; H, 5.43; N, 22.21; found: C, 57.18; H, 5.48; N, 22.20.

#### 3.1.5. The General Procedure for the Synthesis of Compound **8**

A few drops of triethylamine were added to a solution of nicotinoyl-glycyl-glycine-hydrazide (**4**) (20 mmol, 6.24 g) in dioxane (30 mL) with diethylmalonate (20 mmol, 3.2 g). The blend was refluxed for five hours. The resulting solvent was concentrated, and the precipitate filtered and crystallized from ethanol to create compound **8.**

***N-(2-(2-(3, 5-dioxopyrazolidin-1-yl)-2-oxoethylamino)-2-oxoethyl) nicotinamide* (8).** Yield: 65%; m.p. 140–142 °C, IR (cm^−1^): (KBr): ν = 3429 (NH stretching), 3072 (CH, aromatic), 2985 (CH, aliphatic), 1716, 1592, 1491, and 1416 (C=O amide I, II, III and IV, respectively*). ^1^H-NMR (500 MHz, δ, ppm, DMSO-d_6_): δ* = 9.08–8.27 (s, 3H, 3NH, D_2_O exchangeable, amide, hydrazide), 7.57–7.54 (s, 4H, aromatic H, Pyr.), 4.12, 4.07 (t, 4H, 2CH_2_, α-gly), 3.36 (t, 2H, CH_2_, Pyrazole ring). MS (EI, 70 eV): *m/z* (%) = 319 (M^+^, 0.05), 217 (0.05), 137 (1.58), 123 (100), 105 (95.03), 78 (75.36), 50 (26.88). Molecular formula (M.wt.): C_13_H_13_N_5_O_5_ (319.3). Calculated analysis: C, 48.90; H, 4.10; N, 21.94; found: C, 48.94; H, 4.13; N, 21.97.

#### 3.1.6. The General Procedure for Compound **9** Synthesis

A solution of potassium hydroxide (10 mmol, 0.56 g) in ethanol (30 mL) and compound **4** (10 mmol, 4.53 g) was added and well stirred. The stirring was continued for half an hour and then carbon disulfide (2 mL) was added. The blend was refluxed for six hours and the solvent was evaporated under diminished pressure. Then, water (25 mL) was affixed. In an ice bath, the resulting solution was acidified with dilute hydrochloric acid in a drop-like manner. The precipitate was rinsed with water, dried, and crystallized from ethanol to yield the oxadiazole (**9**).

***N-(2-oxo-2-((5-thioxo-4, 5-dihydro-1, 3, 4-oxadiazol-2-yl) methylamino) ethyl) nicotinamide* (9).** Yield: 60%; m.p. 197–199 °C*, IR (cm^−1^): (KBr): ν =* 3429 (NH stretching), 3317 (CH, aromatic), 3194 (CH, aliphatic), 1719 and 1654 (C=O amide I and II, respectively). ^1^H-NMR (500 MHz, δ, ppm, DMSO-*d_6_*): δ = 9.22, 9.00 (s, 3H, 3NH, D_2_O exchangeable, amide, and hydrazide), 8.70–7.80 (s, 4H, aromatic H, Pyr.), 4.00, 3.70 (t, 4H, 2CH_2_, α-gly). MS (EI, 70 eV): *m/z* (%) = 294 (M^+^ + 1, 1.67), 199 (5.77), 105 (10.99), 69 (48.96), 59 (100), 57 (66.25), 55 (71.70), 50 (2.54). Molecular formula (M.wt.): C_11_H_11_N_5_O_3_S (293.3). Calculated analysis: C, 45.04; H, 3.78; N, 23.88; S, 10.93; found: C, 45.01; H, 3.76; N, 23.85; S, 10.90.

#### 3.1.7. The General Procedure for Compounds **10** and **11** Synthesis

Compound **4** (02.11gm, 10 mmole) and ethyl cyanoacetate and/or ethyl acetoacetate (01.33 gm, 10 mmole) were heated under reflux in ethanol (30 mL, dry conditions) for 12 h in a mixture. The mixture was streamed onto crushed ice (10 mL). The solid precipitate was rinsed thoroughly with ethanol and dried to produce compounds **10** and **11,** respectively.

***N-(2-(2-(5-amino-3-hydroxy-1H-pyrazol-1-yl)-2-oxoethylamino)-2-oxoethyl) nicotinamide* (10).** Yield: 85%; m.p. 136–138 °C, IR (cm^−1^): (KBr): ν = 3432 (NH stretching), 2999 (CH, aromatic), 2918 (CH, aliphatic), 1634, 1432, and 1317 (C=O amide I, II and III, respectively). ^1^H-NMR (500 MHz, δ, ppm, DMSO-*d_6_): δ* = 10.5 (s, 1H, OH aromatic, D_2_O exchangeable), 9.3, 9.1 (s, 2H, 2NH, D_2_O exchangeable, amide), 8.7–7.9 (s, 4H, aromatic H, Pyr.), 6.6 (s, 2H, NH_2_), 6.1 (d, 1H, CH, Pyrazole ring), 4.4, 3.9 (t, 4H, 2CH_2_, α-gly). MS (EI, 70 eV): *m/z* (%) = 319 (M^+^ + 1, 0.24), 318 (M^+^, 1.03), 257 (0.56), 189 (1.00), 97 (28.84), 63 (48.28), 59 (100), 55 (66.81), 50 (2.51). Molecular formula (M.wt.): C_13_H_14_N_6_O_4_ (318.3). Calculated analysis: C, 49.06; H, 4.43; N, 26.40; found: C, 49.08; H, 4.44; N, 26.37.

***N-(2-(2-(3-methyl-5-oxo-4,5-dihydro-1H-pyrazol-1-yl)-2-oxoethylamino)-2-oxoethyl) nicotinamide* (11)**. Yield: 80%; m.p. 230–232 °C, IR (cm^−1^): (KBr): ν = 3420 (NH stretching), 3000 (CH, aromatic), 2910 (CH, aliphatic), 1630, 1444, and 1350 (C=O amide I, II and III, respectively). ^1^H-NMR (500 MHz, δ, ppm, DMSO-*d_6_): δ* = 9.4, 9.2 (s, 2H, 2NH, D_2_O exchangeable, amide), 8.8–8.1 (s, 4H, aromatic H, Pyr.), 6.3 (d, 1H, CH, Pyrazole ring), 4.5, 4.2 (t, 4H, 2CH_2_, α-gly), 2.5 (s, 2H, CH_2_, pyrazol moity), 2.0 (s, 3H, CH_3_). MS (EI, 70 eV): *m/z* (%) = 318 (M^+^ + 1, 1.20), 317 (M^+^, 3.11), 212 (5.15), 160 (11.00), 97 (33.99), 63 (58.55), 59 (100), 55 (77.60), 50 (2.11). Molecular formula (M.wt.): C_14_H_15_N_5_O_4_ (317.3). Calculated analysis: C, 52.99; H, 4.76; N, 22.07; found: C, 52.80; H, 4.70; N, 22.00.

### 3.2. Biological Evaluations

#### Anti-Microbial Activity (Agar Diffusion Assay)

The samples were dissolved in dimethyl sulfoxide at a concentration of 1 mg/1 mL to cross-reference of different standard antibiotics. The strain used to test organisms was as follows: A-bacteria, e.g., *Escherichia coli* (ATCC 25922) and *Bacillus subtilis* (NRRL-B-4219) and B-test fungi, e.g., *Aspergillus niger* (ATCC 16888) and *Candida albicans* (ATCC 10231). The anti-microbial activity of newly synthesized compounds was evaluated via an agar disc diffusion assay [52]. The samples were dissolved in distilled water. Briefly, a 24 h old culture of bacteria and 48 h old culture of fungi, respectively. The cultures were mixed with sterile physiological saline (0.9%) and the turbidity was modified to the standard inoculums of the Mac Farl and scale at 0.5 (10^6^ Colony-Forming Units (CFU) per mL). Petri plates containing 20 mL of Mueller Hinton Agar (Lab M., Bury, Lancashire, UK) and Sabouraud-dextrose agar (Lab M., Bury, Lancashire, UK) was used for antibacterial and antifungal activity. The inoculums were spread on the surface of the solidified media and Whatman No. 1 filter paper discs (6 mm in diameter) impregnated with the test compound (40 μL/disc) were placed on the solidified media. Standard bacterial and fungal antibiotics NA = Negram (nalidixic acid), S = Streptomycin; N = Neomycin, NY = Nystatin; T = Oxytetracycline, VA = Vancomycin, CDZ = Cefodizime, NV = Novobiocin were used to positively regulate the bacteria and fungi. Inhibition zones were recorded in millimeters after incubating bacterial strains at 37 °C for 24 h and fungal strains at 25 °C for 72 h. Tests took place in triplicate; the values were presented as mean ± standard deviation (SD) [53].

### 3.3. Computational Studies

#### 3.3.1. Molecular Modeling

##### Protein and Ligand Preparation for Docking Studies

In the preparation of ligands: the MOE-builder tool, which is part of the MOE suit of the MOE version 2014.10 software [54] was used to build the crystal structure; furthermore, the MMFF994x was applied to minimize the energy of these compounds up to the conjugate gradient Root Mean Square (RMS) was less than 0.05 kcal/mol Å^−1^. Moreover, PM3/ESP and AM1 methodology were used to calculate the partial charges and the partial atomic charges for ligands and atoms, respectively [55]. The method of Semi empirical performed in MOE.

In the preparation of macromolecules: The crystal structure of penicillin-binding protein 3 (PBP3) from methicilin-resistant *Staphylococcus aureus* in the cefotaxime bound form (3VSL, Resolution: 2.4 Å) [56] and the crystal structure of sterol 14-alpha demethylase (CYP51) from Trypanosoma brucei bound to an inhibitor *N*-(1-(2,4-dichlorophenyl)-2-(1*H*-imidazol-1-yl)ethyl)-4-(5-phenyl-1,3,4-oxaziazol-2-yl)benzamide (3GW9, resolution 1.87 Å) were acquired from the Protein Data Bank. Thereafter, AMBER10 [57,58] force field and AMBER10 atomic charges [59] were used, after addition of hydrogens atoms to that protein molecules, to minimize the macromolecules energy (RMS force < 0.01 kcal mol^−1^ Å^−1)^ via the method of truncated Newton. Restrained electrostatic potential (RESP) charges based on quantum chemical calculations was used for the heme component charge of CYP51 [60]. The heme group’s iron metal atom was set to three charges. According to the pH of 7.4, the protonation states were assigned using the method “MOE Protonate 3D.” Then Histidine protonation states were assigned in accordance with their H-bonding conditions. The protein’s net charge was 6.0.

The enzyme was designed for the docking studies where: (i) The enzyme’s active site was ligand molecule; (ii) the elimination of water molecules, leaving the water molecules that in active site of binding substrates of the CYP51); (iii) wall restriction was created from the alpha spheres obtained for the active sites search in the structure of enzyme.

##### Non-Covalent Docking Studies

Studies of Docking was carried with software from MOE-Dock, [60] which provides flexibility for side chains. Ligand placement with London dG scoring function was done using the Alpha performance moment integration (PMI) method. The method of Alpha PMI placement achieves poses via the inertia principal moments of aligning ligand conformation to a randomly selected ligands’ subset in site of the receptor. The scoring function, which was used to evaluate binding scoring, was the London dG that is based on the binding-free energy estimation of the ligand from a specified pose [61,62]. Furthermore, the force field energy minimization (MMFF94x) with generalized born solvation model were used to retain and refine for the top 30 poses [61] and the side chain residues of receptor was accepted within 6 Å to relax around the mobile ligand. However, a constant of force of 1.0 kcal mol^−1^·Å^−2^ was applied to the receptor side chains. In addition, when the cutoff value of gradient for the RMS was reached to 0.01 kcal mol^−1^·Å^−2^, the minimization of energy is stopped. Finally, to determine free binding energy of the ranked final poses, the method of GBVI/WSA dG was performed.

##### Covalent Docking Studies

All synthesized and references compounds were covalently docked into the active site of Penicillin-binding protein 3’s (PBP3) using the covalent docking module [63] in MOE, which was used to predict the algorithm for the pose of conformational sampling. The reaction type of covalent bonding was set to be aldehyde acetalization, theoretical assumption, the carbon of carbonyl group in ligand is reacted with hydroxyl group in the Ser392. The refinement of pose and steps of scoring were used with the default settings.

##### ADMET Properties

A computational study of synthesized compounds **4**–**11** took place to estimate the properties of ADMET. In the current study, ADMET predictor Discovery Studio 4.5 was used to evaluate ADMET properties [64]. This tool is used within Insilco ADMET filtering. The synthesized compounds’ compliance with the Lipinski’s rule of five was computed [65]. This approach has been broadly applied to filter for substances that would likely be further developed for drug design programs. Additionally, parameters such as numbers of rotatable bonds (>10) and rigid bonds were evaluated, showing the potential of the compounds to maintain desirable oral bioavailability and intestinal absorption [66].

##### In Silico Toxicity

TOPKAT (Discovery Studio 4.5, Accelrys, Inc., San Diego, CA, USA), an established software tool for prediction of toxicity in silico, was used to anticipate the potential toxicity of all compounds. TOPKAT uses sharply devised and verified QSTR models to anticipate the probability values of toxicity. The QSTR models in TOPKAT are devised by regression analysis for perpetual endpoints while using discriminate analysis for categorical endpoints. TOPKAT efficiently uses various two-dimensional molecular, spatial, and electronic indicators to devise the models of prediction.

To gauge the validity of the prediction, TOPKAT uses the patented OPS for method validation. Values of probability less than 0.30 are established as low toxicological endpoint probabilities. On the other hand, values exceeding 0.70 are deemed as high probabilities [44].

## 4. Conclusions

In the current study, some novel dipeptide candidates (**5**–**11**) were devised. The reaction of the coupling of nicotinic acid with a certain L-amino acid methyl ester took place via solution–phase peptide synthesis. All newly synthesized compounds were fully evaluated by spectroscopic data and validated for purity. The biological activity of the novel dipeptide derivatives was evaluated, which showed different anti-microbial inhibitory activities based on the type of pathogenic microorganisms. The anti-microbial activities taking place in vitro of the novel candidates were crosschecked against Gram-positive and Gram-negative bacteria, yeast and filamentous fungi, *Bacillus subtilits*, *Escherichia coli, Candida albicans*, and *Aspergillus niger*, respectively. Some of the assayed compounds exhibited the strongest antibacterial activities. The crosscheck against Gram-positive and Gram-negative bacteria and fungi is a good indicator for novel dipeptide derivatives that can be used in the treatment of Gram-positive and Gram-negative bacteria and fungus pathogens or other compounds with an effect expanding on a wide spectrum. Followed by molecular docking and interpretation, it was detected that the newly Nicotinoylglycine derivatives formed an H-bond in non-covalent docking with Ser448, Glu623, Gln524, Thr62, Thr619, and Asn450 of Penicillin-Binding Protein 3 (PBP3) (3VSL), while it formed an H-bond with Met106, Tyr116, Ala287, Met460, Met 358, Leu357, Met360, and HEM480 of Sterol 14-Alpha Demethylase (CYP51) (3GW9), whereas it formed a covalent bond with Ser392 and H-bond with Ser392, Thr603, Ser448, Asn450, Ser448, Thr621, and Pro660 of Penicillin-Binding Protein 3 (PBP3) (3VSL), although it is clearly understood that the novel compound **4**, **7**, **8**, **9** and **10** according to the binding interaction and biological activities have higher inhibitory efficacy towards (PBP3) and (CYP51). Furthermore, based on the docking score and binding affinity, compounds with the least binding affinity and docking score values were selected to be highly potential drugs. Analysis of the ADMET parameters for the synthesized compounds **4**–**11** showed their optimal oral drug-like traits in most compounds and the potential for development as candidates for oral drugs. In addition, the cytotoxicity prediction of all compounds is minimal, and they retain a high safety profile, indicating anti-microbial action selectivity in them.

## Figures and Tables

**Table 1:** The anti-microbial activities of the newly synthesized candidates expressed as inhibition zones of growth in mm against the used test organisms.

Compounds	Test Organism			
	Bacteria		Fungi	
	Gram-Positive	Gram-Negative	Unicellular	Filamentous
	*B. subtilits*	*E. coli*	*C. albicans*	*A. niger*
**Inhibition Zone (mm)**				
4	29	30	28	16
5	15	00	17	00
6	15	13	16	00
7	18	15	20	00
8	15	14	20	16
9	19	15	20	00
10	11	15	15	00
11	11	12	15	00
**Standard Antibiotics**				
NA = 30 µg	20	16	00	00
S = 10 µg	14	00	12	00
N = 30 µg	00	00	00	16
Ny = 100 µg	00	00	00	00
NV = 30 µg	29	30	00	00
T = 30 µg	30	27	00	00
SDZ = 30 µg	00	20	00	00
VA = 30 µg	21	23	00	00

Standard bacterial antibiotics, NA = Nalidixic Acid (Negram), S = streptomycin; N = neomycin, Ny = nystatin; VA = T = oxytetracycline, vancomycin, CDZ = Cefodizime, NV = novobiocin.

**Table 2:** Predicted binding-free energies (ΔGbind) for ligands **4**–**11**.

Receptor	Compound	Amino Acid Residues	Type of Interaction	Distance (Å)	Binding Affinity kcal·mol^−1^	Docking Score (S)	RMSD Refine
Antibacterial Penicillin-Binding Protein 3 (PBP3) (3VSL)	Compound **4**	Ser448	H-donor	2.40	−5.315	−11.868	2.258
		Glu623	H-acceptor	2.36			
		Gln524	pi-H	2.80			
	Compound **5**	Thr621	H-donor	1.87	−6.177	−10.463	2.559
		Thr619	H-acceptor	1.82			
		Thr621	H-acceptor	2.47			
		Thr621	H-acceptor	193			
	Compound **6**	Thr621	H-acceptor	2.83	−6.228	−10.831	2.610
		Gln524	Pi-H	3.27			
	Compound **7**	Glu623	H-acceptor	2.70	−5.799	−10.280	1.658
		Asn450	H-acceptor	2.10			
		Gln524	Pi-H	2.86			
	Compound **8**	Glu623	H-acceptor	2.37	−5.822	−10.287	2.194
		Thr621	H-acceptor	2.09			
		Thr621	H-acceptor	2.57			
		Gln524	Pi-H	2.87			
	Compound **9**	Glu623	H-donor	1.99	−5.508	−10.891	2.741
		Ser448	H-donor	2.54			
		Thr621	H-acceptor	3.16			
		Thr621	H-acceptor	2.85			
		HOH894	Pi-H	2.75			
	Compound **10**	Glu623	H-donor	2.59	−5.790	−9.695	1.625
		Thr621	H-acceptor	2.00			
		Thr621	H-acceptor	2.24			
	Compound **11**	Glu623	H-donor	3.06 3.22	−5.47	−10.372	1.5346
		Thr621	H-donor				
		Gln524	pi-H				
	Standard drug S	Val658	H-donor	3.12	−7.152	−17.891	1.982
		Glu623	H-donor	2.9			
		Thr621	H-donor	3.48			
		Thr621	H-acceptor	3.06			
		Asn450	H-acceptor	3.1			
		Asn450	H-acceptor	2.9			
		Glu623	ionic	2.9			
		Glu623	ionic	3.77			
		Ser448	ionic	4.03			
	Standard drug NA	Ser429	H-acceptor	2.84	−4.781	−9.597	0.551
		Asn450	H-acceptor	3.11			
	Standard drug N	Asp601	H-donor	3.17	−6.739	−10.590	2.123
		His447	H-donor	3.21			
		Thr621	H-donor	3.44			
		Asn450	H-donor	3.12			
		Glu623	H-donor	2.86			
		Glu623	H-donor	3.43			
		Glu623	ionic	2.86			
		Glu623	ionic	3.43			

**Table 3:** Predicted binding-free energies (ΔGbind) for ligands **4**–**11**.

Receptor	Compound	Amino Acid Residues	Type of Interaction	Distance (Å)	Docking Score (S)	Binding Affinity kcal·mol^−1^	RMSD Refine
Antifungal Sterol 14-Alpha Demethylase (CYP51) (3GW9)	Compound **4**	Met106 Met460 HOH566	H-donor Pi-H H-acceptor	2.90 3.53	−9.333	−5.886	2.145
	Compound **5**	Met 358	Pi-H	3.34	−9.690	−5.674	1.693
		HOH546	H-acceptor	1.79			
	Compound **6**	Met106	H-donor	4.82	−9.531	−6.132	3.165
		Leu357	H-acceptor	2.63			
	Compound **7**	Met106	H-donor	4.47	−9.043	−5.940	2.008
		Met358	pi-H	3.95			
	Compound **8**	Met106	H-donor	3.44	−10.359	−6.686	2.01
		Leu357	H-acceptor	1.87			
		HEM480	ionic	2.85			
	Compound **9**	Met106	H-donor	3.67	−9.401	−6.157	1.042
		Tyr116	H-donor	2.01			
		Ala287	H-acceptor	3.20			
		Met460	Pi-H	3.53			
		HEM480	Pi-H	2.89			
	Compound **10**	Met106	H-donor	3.43	−9.884	−5.783	2.341
		Leu357	H-acceptor	1.97			
		HEM480	H-pi	2.95			
	Compound **11**	Met106	H-donor	3.62	−9.503	−5.407	1.176
	Standard drug S	Met360	H-donor	2.89	−12.996	−5.573	2.291
		Met358	H-acceptor	3.16			
	Standard drug NA	Ala291	pi-H	4.18	−10.169	−6.329	1.145
	Standard drug N	Met106	H-donor	3.7	−11.891	−6.792	2.75
		Tyr116	H-donor	2.92			

**Table 4:** Predicted covalent-binding-free energies (ΔGbind) for ligands **4**–**11**.

Receptor	Compound	Amino Acid Residues	Type of Interaction	Distance (Å)	Binding Affinity Kcal/moL	Docking Score (S)	RMSD Refine
Antibacterial Penicillin-Binding Protein 3 (PBP3) (3VSL)	Compound **4**	Ser392	Covalent bond		−4.315	−10.254	2.258
		Asn450	H-acceptor	1.99			
		Ser448	H-acceptor	2.31			
		Thr621	H-donor	2.32			
		Pro660	Pi-H	3.19			
		HOH883	H-acceptor	2.21			
	Compound **5**	Ser392	Covalent bond		−5.031	−9.73	1.322
		Thr621	H-acceptor	2.56			
		Glu623	H-donor	1.96			
	Compound **6**	Ser392	Covalent bond		−5.966	−9.94	0.6999
		Thr603	H-donor	2.08			
	Compound **7**	Ser392	Covalent bond		−4.782	−9.466	1.472
		Asn450	H-acceptor	2.13			
		Thr621	H-donor	2.13			
	Compound **8**	Ser392	Covalent bond		−5.73	−9.161	0.7531
		Asn450	H-acceptor	2.12			
		Thr621	H-acceptor	2.14			
		Thr621	H-acceptor	2.38			
		Ser448	pi-H	3.89			
	Compound **9**	Ser392	Covalent bond	1.60	−4.97	−9.234	1.012
		Thr621	H-acceptor	1.83			
		Thr621	H-acceptor	2.04			
		Asn450	H-donor	3.10			
	Compound **10**	Ser392	Covalent bond		−5.602	−11.84	0.8385
		Thr621	H-acceptor	2.05			
		Thr621	H-acceptor	2.27			
		Asn450	H-acceptor	2.22			
		Ser448	Pi-H	2.91			
	Compound **11**	Ser392	Covalent bond		−5.777	−9.822	1.155
		Thr621	H-acceptor	2.06			
		Thr621	H-acceptor	2.26			
		Asn450	H-acceptor	2.26			
		Ser448	Pi-H	2.83			
	NA	Ser392	Covalent bond		−4.528	−7.344	1.003
		Thr621	H-donor	2.02			

**Table 5:** Absorption, distribution, metabolism, and excretion (ADMET) prediction of all compounds.

Name	Comp. 4	Comp. 5	Comp. 6	Comp. 7	Comp. 8	Comp. 9	Comp. 10	Comp. 11
**Formula**	C_10_H_13_N5O3	C_15_H_21_N_5_O_7_	C_16_H_23_N_5_O_8_	C_15_H_17_N_5_O_3_	C_13_H_13_N_5_O_5_	C_11_H_11_N_5_O_3_S	C_13_H_14_N_6_O_4_	C_14_H_15_N_5_O_4_
MWt (≤450 g/mol)	251.24	383.36	413.38	315.33	319.27	293.3	318.29	317.3
Heavy Atoms	18	27	29	23	23	20	23	23
Aromatic Heavy Atoms	6	6	6	11	6	11	11	6
Rotatable Bonds (Nrot < 10)	8	13	14	8	8	7	8	8
H-bond Acceptors (HBA ≤ 8)	5	9	10	5	6	5	6	6
H-Bond Donors (HBD ≤ 5)	4	7	8	2	3	3	4	2
The distribution coefficient (LogD 3.5)	2.588	4.217	4.728	0.675	2.332	0.488	1.428	2.037
the topological polar surface area (TPSA ≤ 140)	126.2	193.47	213.7	105.98	137.57	145	152.23	120.83
LogPo/w	0.41	0.06	0.91	2.09	0.14	1.36	1.25	1.55
log Kp (cm/s)	−9.2	−11.29	−11.92	−8.09	−9.53	−8.34	−8.54	−9.11
Lipinski Violations	0	2	2	0	0	0	0	0
Ghose Violations	1	1	1	0	1	0	1	1
Bioavailability Score	0.55	0.17	0.17	0.55	0.55	0.55	0.55	0.55
PAINS Alerts	0	0	0	0	1	0	0	0
Brenk Alerts	2	1	1	0	1	1	0	0
Solubility (logS ≥ 0.010 mg/mL)	3.16$\times 10^{2}$	$2.61\times 10^{3}$	1.26$\times 10^{3}$	5.73$\times 10^{0}$	1.42$\times 10^{2}$	1.21$\times 10^{1}$	1.34$\times 10^{1}$	6.37$\times 10^{1}$
Solubility Level	5	5	5	4	4	4	4	4
Unknown, AlogP98	1	0	0	0	2	0	0	1
EXT_PPB	−8.478	−13.247	−15.348	−5.4949	−8.288	−8.3825	−9.958	−7.986
AlogP98	−2.076	−4.217	−4.728	−0.675	−1.819	−0.611	−1.428	−1.525
BBB Level	4	4	4	4	4	4	4	4
	undefined	undefined	undefined	undefined	undefined	undefined	undefined	undefined
EXT Hepatotoxic	0.427	-1.967	-2.013	2.865	-0.2698	2.3875	3.387	0.8041
Absorption Level	2	3	3	0	2	0	3	1
	Low	Very low	Very low	very good	Low	very good	Very low	moderate
CYP2D6	false	false	false	false	false	false	false	False
Hepatotoxic	true	true	true	true	true	true	true	true
PPB	false	false	false	false	false	false	false	false

**Table 6:** Probability values of different toxicity models of compounds **4**–**11** by TOPKAT analyze.

Name	Comp. 4	Comp. 5	Comp. 6	Comp. 7	Comp. 8	Comp. 9	Comp. 10	Comp. 11
Mouse Female NTP **	0.575	0.527	0.502	0.571	0.57	0.599	0.516	0.501
Mouse Male NTP	0.576	0.247	0.189	0.539	0.578	0.469	0.546	0.403
Rat Female NTP	0.478	0.381	0.391	0.462	0.446	0.482	0.417	0.318
Rat Male NTP	0.353	0.2	0.222	0.348	0.384	0.385	0.319	0.237
Mouse Female FDA * None vs. Carcinogen	0.209	0.206	0.205	0.205	0.21	0.218	0.205	0.207
Mouse Male FDA None vs. Carcinogen	0.294	0.198	0.187	0.334	0.259	0.281	0.275	0.296
Rat Female FDA None vs. Carcinogen	0.273	0.226	0.225	0.292	0.283	0.307	0.3	0.291
Rat Male FDA None vs. Carcinogen	0.294	0.299	0.324	0.366	0.313	0.328	0.367	0.324
Mouse Male FDA Single vs. Multiple	0.162	-	-	0.148	-	0.185	0.149	0.161
Rat Female FDA Single vs. Multiple	0.529	-	-	0.55	0.53	0.583	0.566	0.533
WOE	0.469	0.384	0.368	0.475	0.471	0.461	0.477	0.434
Ames	0.621	0.495	0.454	0.533	0.521	0.684	0.581	0.544
DTP	0.567	0.573	0.607	0.485	0.503	0.507	0.502	0.501
Skin Irritancy None vs. Irritant	0.972	0.973	0.973	0.973	0.972	0.974	0.962	0.974
Skin Sensitization None vs. Sensitizer	0.669	0.545	0.572	0.612	0.659	0.744	0.629	0.658
Ocular Irritancy None vs. Irritant	0.999975	0.999942	0.999942	0.999976	0.999967	0.999988	0.999975	0.999977
Ocular Irritancy Mild vs. Moderate Severe	0.836	0.891	0.891	0.838	0.866	0.834	0.834	0.83
Ocular Irritancy Moderate vs. Severe	0.663	0.662	0.665	0.69	0.645	0.668	0.613	0.636
Aerobic Biodegradability	0.472	0.648	0.653	0.548	0.55	0.439	0.318	0.563

* FDA: Food and Drug Administration; NTP **: National Toxicology Program.
